# GAN-enhanced deep learning for improved Alzheimer's disease classification and longitudinal brain change analysis

**DOI:** 10.3389/fmed.2025.1587026

**Published:** 2025-06-17

**Authors:** Purushottam Pandey, Surbhi Bhatia Khan, Jyoti Pruthi, Eid Albalawi, Ali Algarni, Ahlam Almusharraf

**Affiliations:** ^1^Manav Rachna University (MRU), Faridabad, Haryana, India; ^2^School of Science, Engineering and Environment, University of Salford, Salford, United Kingdom; ^3^Centre for Research Impact & Outcome, Chitkara University, Institute of Engineering and Technology, Rajpura, Punjab, India; ^4^Research and Innovation Cell, Rayat Bahra University, Mohali, Punjab, India; ^5^Department of Computer Science, College of Computer Sciences and Information Technology, King Faisal University, Al Ahsa, Saudi Arabia; ^6^Department of Informatics and Computer Systems, College of Computer Science, King Khalid University, Abha, Saudi Arabia; ^7^Department of Management, College of Business Administration, Princess Nourah Bint Abdulrahman University, Riyadh, Saudi Arabia

**Keywords:** Alzheimer's disease, ResNet101, long short term memory, generative adversarial network, ADNI, OASIS dataset

## Abstract

Alzheimer's disease (AD) is commonly defined by a progressive decline in cognitive functions and memory. Early detection is crucial to mitigate the devastating impacts of AD, which can significantly impair a person's quality of life. Traditional methods for diagnosing AD, while still in use, often involve time-consuming processes that are prone to errors and inefficiencies. These manual techniques are limited in their ability to handle the vast amount of data associated with the disease, leading to slower diagnosis and potential misclassification. Advancements in artificial intelligence (AI), specifically machine learning (ML) and deep learning (DL), offer promising solutions to these challenges. AI techniques can process large datasets with high accuracy, significantly improving the speed and precision of AD detection. However, despite these advancements, issues such as limited accuracy, computational complexity, and the risk of overfitting still pose challenges in the field of AD classification. To address these challenges, the proposed study integrates deep learning architectures, particularly ResNet101 and long short-term memory (LSTM) networks, to enhance both feature extraction and classification of AD. The ResNet101 model is augmented with innovative layers such as the pattern descriptor parsing operation (PDPO) and the detection convolutional kernel layer (DCK), which are designed to extract the most relevant features from datasets such as ADNI and OASIS. These features are then processed through the LSTM model, which classifies individuals into categories such as cognitively normal (CN), mild cognitive impairment (MCI), and Alzheimer's disease (AD). Another key aspect of the research is the use of generative adversarial networks (GANs) to identify the progressive or non-progressive nature of AD. By employing both a generator and a discriminator, the GAN model detects whether the AD state is advancing. If the original and predicted classes align, AD is deemed non-progressive; if they differ, the disease is progressing. This innovative approach provides a nuanced view of AD, which could lead to more precise and personalized treatment plans. The numerical outcome obtained by the proposed model for ADNI dataset is 0.9931, and for OASIS dataset, the accuracy gained by the model is 0.9985. Ultimately, this research aims to offer significant contributions to the medical field, helping healthcare professionals diagnose AD more accurately and efficiently, thus improving patient outcomes. Furthermore, brain simulation models are integrated into this framework to provide deeper insights into the underlying neural mechanisms of AD. These brain simulation models help visualize and predict how AD may evolve in different regions of the brain, enhancing both diagnosis and treatment planning.

## 1 Introduction

AD (Alzheimer's disease) is one of the leading causes of dementia universally (1, 2) and considered as one of the most deadly diseases which needs to be taken under consideration with utmost care. AD is characterized by a recurrent deterioration of cognitive abilities in older people. Besides, AD is stated as an irreversible neurological disorder which progressively impairs the cognitive capability therefore, it is important to provide effective treatments as early as possible with the aim to avoid life threatening consequences. It was reported that, AD is expected to rise from 27 to 106 million cases (3) in the upcoming four decades, impacting one in every 85 people on the planet. Another report suggested that ~70% people are account of AD (4). As there is an evident rise of AD in recent times, effective methods need to be implemented for detection of AD in people; however, to treat AD, it is important to identify the symptoms in the patients suffering with AD; thus, some of the common symptoms of people suffering with AD are memory loss, difficulty in speaking, loss of spontaneity, and many more (5–7). Usually, people with AD can endure symptoms for years; however, the severity of AD symptoms tends to worsen progressively, gradually impairing an individual's ability to perform everyday activities independently. Since there is currently no known cure for AD, nevertheless existing treatments aim to slow down the disease's advancement and delay the onset of its most severe stage.

Typically, AD is classified into three stages such as mild, moderate, and severe (8). Early stages of AD can perform daily tasks independently, although they may struggle with specific tasks (9) such as driving, individuals in early stage can communicate socially and remember significant details. However, as the disease progresses to the middle stage, symptoms become more pronounced and the person may require greater care, frustration, and difficulty with routine tasks (10, 11). In the last stage, AD becomes the most challenging for managing as individuals lose their ability to respond and communicate leading to a significant decline in memory and cognitive skills (12, 13). Therefore, it is extremely important to detect the symptoms as early as possible with the aim to avert any impemending consequences faced by the individuals. Hence, different manual techniques are primarily used by the medical professionals for AD detection which includes cognitive assessments and neurological examinations where healthcare providers assess the functions and activities of brain to detect any abnormalities which may be indicative of AD. Furthermore, brain imaging techniques such as PET scans and MRI are used for providing detailed images of the brain to medical experts. Although these techniques offer various advantages, there are certain drawbacks of employing manual techniques (13, 14) such as time-consuming, subjectivity, and prone to error, which require highly skilled medical professionals. Hence, to overcome these drawbacks faced by manual approaches, AI-based techniques are incorporated as AI-based models are fast and accurate and can handle huge amount of complex data easily. Moreover, AI models can detect any subtler changes in functioning of brain which may not be easily detectable by human observers. Hence, various existing research study focuses on employing AI-based ML and DL models for the detection and classification of AI.

Dense neural network is used for binary classification of Alzheimer's disease by alleviating the problem of multiple modalities and processes. A fully connected dense neural network (FCNN) with two hidden layers (15) was used for performing binary classification of AD. By applying FCNN model, the accuracy gained by the model is 87.50%. Similarly, CNN-based DL model (16) has used for AD classification using ADNI dataset. In CNN model, different layers such as three convolutional layer, max pooling layer, and fully connected layer are used for classification. Existing study has considered classifying three different classification of AD, which includes AD vs. NC, AD vs. MCI, and MCI vs. NC. Approximately 450 MRI images were used. Process carried out includes pre-processing the images and classifying the obtained pre-processed images. Skull striping, segmentation, registration, and outlining the ROI were some of the pre-processing techniques used for pre-processing the images. The accuracy obtained for three binary classification task with spike pre-training technique was 90.15%, 87.30%, and 83.90%. However, the accuracy obtained by three binary classification without spike was 86%, 83%, and 76%. Therefore, the incorporation of ANN for extracting the relevant features of AD helped in satisfactory classification of AD (17).

**Figure 1:**
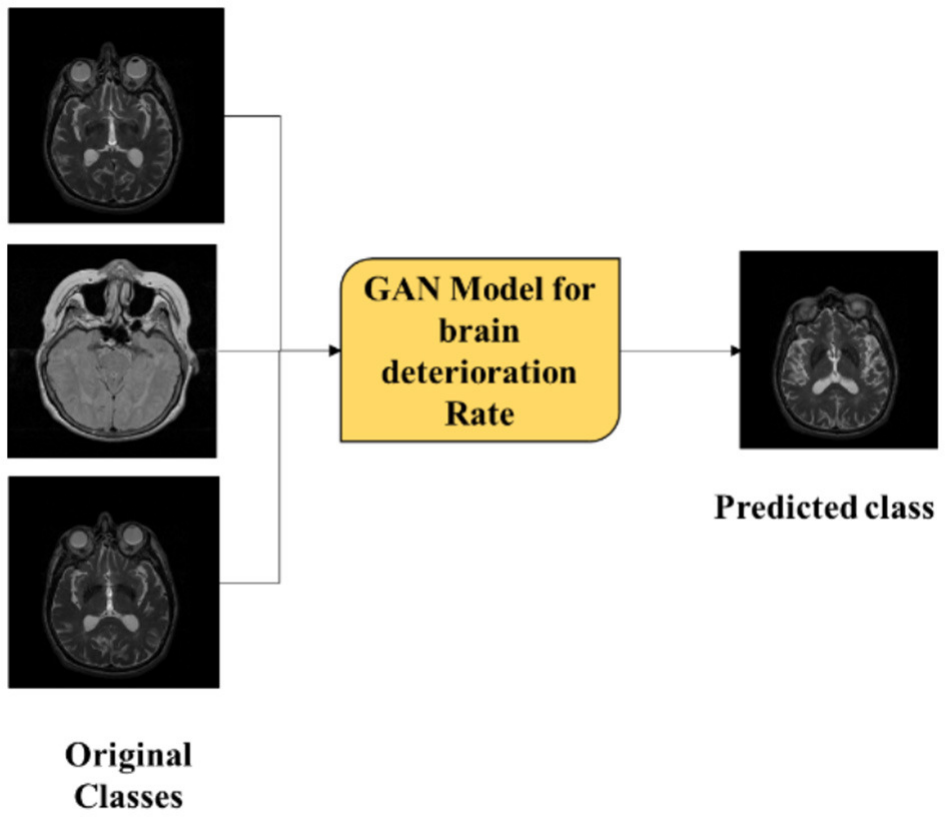
Brain deterioration rate using GAN.

Although the existing models deliver better performance in terms of classification of AD, there are certain pitfalls which need to be addressed. Thus, some of the drawbacks are low accuracies projected by the model, overfitting of the model, empathizing only on binary classification, computational complexity, and inability to work with huge datasets. Thus, to overcome these drawbacks, the proposed model utilizes ResNet101 with LSTM for feature extraction and classification using ADNI and OASIS datasets. The proposed ResNet101 model uses DKCL and PDPO layers to extract relevant features needed for the proposed model. PDPO is employed for assigning binary codes to pixels depending on the comparison with neighboring pixels, by efficiently capturing the local texture information and the DCK layer captures the discriminative effectively by sliding a tiny filter over the input image and computing element-wise multiplication between the filter and overlapping regions of the input data. Implementation of these proposed functions in the proposed ResNet101 model aids in extracting relevant features needed for the model. Eventually, the extracted features are passed to the LSTM model for classification of Alzheimer's disease as AD, CN, and MCI. In addition, the proposed research focuses on employing the GAN model to find whether Alzheimer's disease is progressive or non-progressive in nature by distinguishing the original class from the predicted class. By doing so, the brain deterioration rate can be determined, and this can assist the medical experts to offer a suitable diagnosis to the patients. Thus, Figure 1 depicts the original and predicted class gained using the GAN model, where if the original class and predicted class are the same, it is denoted as non-progressive and if it is different, then it is represented as progressive.

The major contributions of the proposed research study are as follows:

- To extract relevant features for feature extraction using the proposed ResNet101 using pattern descriptor parsing operation layer and detection convolutional kernel layer and to perform multiclass classification using the LSTM model for classifying Alzheimer's disease as CN, MCI, and AD.
- To determine the deterioration rate of the brain as progressive and non-progressive using the proposed model.
- To assess the performance of the proposed model using standard metrics such as accuracy, precision, recall, and F1 score as well as brain deterioration of patients.

The study is organized in the following way. Section 2 deals with existing studies done by research authors. Section 3 discusses proposed algorithms implemented for the classification of AD, Section 4 reflects on the outcome obtained using proposed methodology, and Section 5 summarizes the research study, including future recommendations.

## 2 Literature review

The existing section reviews various existing studies on the detection and classification of Alzheimer's disease using AI-based techniques.

Hybrid DL approach (18) has been used in the study for early Alzheimer's disease detection. Thus, multi-modal imaging and CNN with LSTM algorithm have combined together for identifying early MCI diseases, which remain challenging due to the difficulty in discriminating patients with cognitive normality. Better accuracy was obtained by the model for AD classification. Despite the remarkable performance of the model, the limitation of the model includes overfitting of data. Similarly, two different NNs such as ResNet50 and AlexNet (19) were used for AD detection and classification. The MRI images were collected from Kaggle website, and CNN algorithm was employed using AlexNet and ResNet50 TL models. Accuracy of the model obtained using AlexNet was 94.53%, showcasing that DL model is better suited for medical investigation such as AD detection and classification. Similarly, 12-layer CNN model (20) has been used for AD detection based on brain MRI images. 12-layer CNN model was used on OASIS dataset in which sufficient accuracy has gained by the model for AD classification. Furthermore, the model was compared with other models such as InceptionV3, Xception, MobileNetV2, and VGG19. Although the model has delivered better accuracy for AD classification, the drawback of the model is that it only focused on binary classification of Alzheimer's disease on OASIS dataset.

LSTM (21) has been used for precise diagnostic approach for binary classification of AD. LSTM model was utilized for classifying the MRI data and making accurate predictions for the early detection of AD. Although the model has delivered better performance for binary classification of AD, there are certain drawbacks of the study which needs to be overcome such as inability of the model to fully capture the complexity and variety of the target population. This pitfalls ultimately impact the generalizability and robustness of the model for AD classification. Similarly, LSTM (22)-based RNN model has been used for predicting the progression of the ADF patients from MCI to AD. The objective of the study was to anticipate the development of the illness. LSTM-based model has implemented for predicting the biomarker values using ADNI dataset. The ADNI dataset incorporated the positive biomarker of parents after every 6, 12, 18, 24, and 36 months from the standard. Eventually, the state of progression was identified by using MLP model, where accuracy of 88.24% is accomplished. This findings helped in improving the early findings of AD. Similarly, 3D convolutional and LSTM (ConvLSTM) (23) model has adopted for early diagnosis of AD from full-resolution sMRI scans. Complete resolution of brain images belonging to ADNI and OASIS dataset has been used, in which the accuracy gained by the model is 86%, and F1 score and sensitivity obtained by the model are 88% and 96%. Regardless of the extensive performance of the model, accuracy attained by the model is considerably low.

OASIS dataset (24) has incorporated for identification of AD using DL and image processing approaches. CNN-based DL model has implemented for AD classification, and the accuracy obtained by the model is 93%. Despite its performance, limitation of the model showcases the usage of additional dataset such as ADNI dataset for more comparative validation analysis and tests the generalization of the study. As the model lacks in terms of working with multiple dataset, the future work of the study focuses on creating a bigger dataset combined from different sources for increasing the variability of the input samples of various target class for accomplishing better model in terms of generalization and reliability of the model to new and unseen data. Similarly, CNN-based MobileNet (25) model has been used for multiclass classification of AD, where MobileNet architecture used depthwise separable convolutions that reduced the number of parameters when compared to conventional convolutions and resulted in lightweight neural network. Although the model has delivered better accuracy, different techniques such as augmentation approaches are focused in the future for further enhancing the accuracy of the model.

Two-stage DL model (26) has employed for integrating the process of classification and regression to determine whether a patient is suffering with MCI and then determining the probable progression time. The first stage focused on detecting the patient class using LSTM classification, and the second stage focused on prediction using LSTM regression model. Furthermore, the model was compared with existing ML models such as SVM, RF, LR, KNN, DT Lasso, and Ridge, from which it was identified that suggested LSTM model has delivered better outcome than existing models. In spite of its result, the model lacks in interpreting the decision in an effective way. Thus, the shortcoming of the model includes explainability, accountability, and fairness of the model. CNN-based DL approach (27) has implemented in the study for AD classification, in which the process was carried out by loading OASIS and MIRIAD dataset. Then, CNN has employed for classifying the presence of AD. From the analytical outcome, it was identified that accuracy obtained by CNN model was 82%. Furthermore, sensitivity and specificity gained by the model were 93 and 81%. An 8-layer CNN model called CNN-BN-DO-DA has employed for (28) AD classification in which batch normalization and dropout functions BN was used for normalizing the inputs of the layer into mini-groups in order to solve concerns related to incessant training change and dropout function was utilized for lessening the problems associated with overfitting an computational consumption. OASIS dataset was used. The result of the study has indicated that better techniques will be used in the future for speeding up convergence rate and will be aided in improving the efficacy of the model.

Like DL models, ML models are also used for detecting AD; thus, methods such as DT, SVM, RF, voting classifiers, and gradient boosting (29) were incorporated in the study for identifying the best parameters for AD detection using OASIS dataset. It was detected that accuracy gained by DT, RF, SVM, XGBoost, and voting classifiers was 80.46%, 86.92%, 81.67%, 85.92%, and 85.12%. Although these ML techniques were focused on reducing risks by detecting the disease in early stages, identifying relevant attributes (feature extraction) for the model for AD detection is still challenging task. Bias in ML is an issues which needs to be resolved as quickly as possible; this study (30) has employed Adaptive Synthetic Sampling (ADASYN) technique for improving the accuracy and issues associated with bias. Therefore, feature extraction battery (FEB) and SVM model were employed for feature extraction and classification of AD. It was identified that SVM model has aided in improving the accuracy by 6%. Although the model has obtained better accuracy for AD prediction, ML models along with meta-heuristic approaches were considered in future for further enhancements in terms of improving the prediction accuracy.

Conversely, as stated (31), has aimed to enhance AD classification using MRI data by integrating advanced DL models for early diagnosis and personalized treatment. The method has combined an ensemble DL model with Soft-NMS-enhanced Faster R-CNN for candidate merging, improved ResNet50 for feature extraction, and Bi-GRU for processing sequence data. Using MRI datasets, the model has achieved better classification accuracy for AD vs. CN tasks, demonstrating its potential for precise early diagnosis and intervention. Another study (32) has employed CycleGAN for synthetic image generation and Google Inceptionv3-based CNN for classification. It has utilized CNNs trained on augmented datasets, achieving an F-1 score of 89% with standard data and 95% with CycleGAN-enhanced data augmentation. This approach has shown the effectiveness of DL models and generative adversarial networks in improving diagnostic accuracy for Alzheimer's disease. The author in Zhang et al. (33) has developed ADNet, based on the VGG16 model. It has utilized 2D MRI slices, incorporating depthwise separable convolution, ELU activation, and SE modules for efficient feature extraction while simultaneously training on auxiliary tasks such as clinical dementia and mental state score regression. The findings have shown ADNet achieved a 4.18% accuracy improvement for AD vs. CN classification and a 6% improvement for MCI vs. CN classification compared to the baseline VGG16 model, demonstrating its potential for early diagnosis. Another study (34) has leveraged the ResNet50V2 DL model for AD classification using 6,400 MRI images sourced from Kaggle, achieving a high accuracy of 96.18%. By employing transfer learning, fine-tuning, and dynamic learning rate adjustments, the model effectively discriminated AD stages, which showcased its potential for real-world medical applications. As illustrated (35), has introduced AlzhiNet, a hybrid DL framework that combined 2D-CNN and 3D-CNN models with custom loss functions and volumetric data augmentation for AD diagnosis. It has been validated on MRI datasets, and it has achieved remarkable accuracy and demonstrated robustness against perturbations, outperforming standalone models and ResNet-18 in real-world applications.

### 2.1 Gaps identified

A significant research gap exists in Alzheimer's disease classification using binary models, particularly when addressing challenges associated with small datasets, time consumption, scalability, and overfitting. Current approaches often rely on large datasets to prevent overfitting and ensure robust feature extraction, but neuroimaging studies typically involve limited sample sizes, such as datasets with fewer than a thousand participants or even fewer in some cases. This scarcity leads to difficulties in training deep learning models effectively and exacerbates overfitting risks, especially when high-dimensional data such as MRI scans are involved. In addition, binary classification tasks require discriminative feature selection from complex neuroimaging data, which is computationally demanding and time-consuming. Scalability remains a pressing issue as models optimized for small datasets may not generalize well to larger or diverse populations. Thus, overcoming these limitations requires innovative methodologies that balance computational efficiency with the ability to extract meaningful features from small datasets while mitigating overfitting through advanced regularization techniques or ensemble methods.

## 3 Proposed methodology

**Figure 2:**
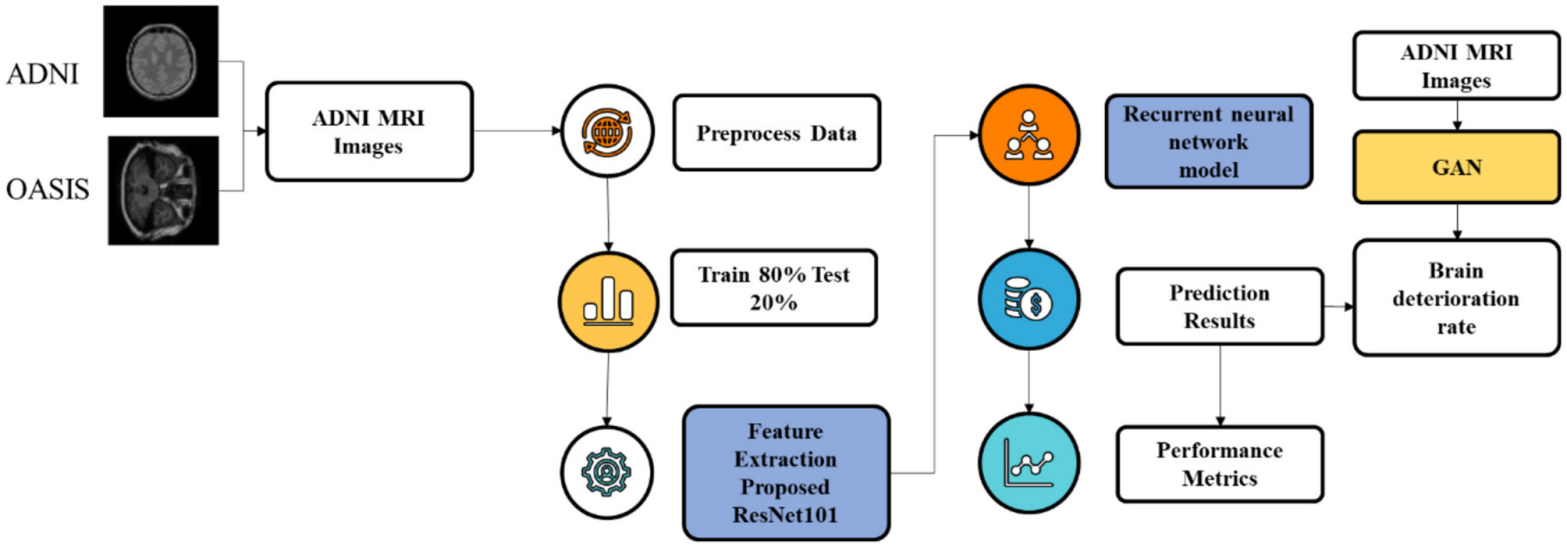
Overall flow of the model.

AD is considered one of the most deadly diseases in the world. Hence, it is important to detect it as quickly as possible. Various approaches are carried out by the research workers. However, there are certain pitfalls of employing existing studies, such as overfitting of the model, low accuracy, computational complexity, and ineffective multiclass classification of AD. Hence, the proposed model is used to overcome these limitations by using efficient algorithms. Thus, the flow of the proposed research is depicted in Figure 2.

Figure 2 depicts the process involved in the proposed research study for multiclass classification of AD. The process is initiated by loading the ADNI and OASIS datasets. Then, the images are pre-processed using image resizing and image data normalization. Image resizing refers to the process of varying the dimensions and resolutions. Thus, resizing the images can aid in standardizing the input data for further processing and analysis. In proposed study, the image is resized in terms of 64 × 64. Image data normalization involves scaling the pixel values to a common range to improve the performance of the model within the range of 0–1. Normalizing image data assists in reducing the variations in pixel intensity and enhances the ability of the model to learn relevant features from the images. Owing to these factors, image resizing and image data normalization are opted for pre-processing. After pre-processing, pre-processed data are split as a train-test split, where the ratio involved in the proposed research for the train-test split is 80:20. After data split, the data are passed onto the proposed ResNet101 and LSTM for feature extraction and classification.

After pre-processing, the proposed ResNet101 is used for feature extraction by employing DKCL and PDPO functions for extracting relevant features. Then, LSTM is employed to classify the images as CN, MCI, and AD accordingly. Eventually, the present research study focuses on determining the deterioration rate of the brain by using the GAN model. This GAN model shows if the disease is in a progressive or non-progressive state by comparing the original class and predicted class. If the original class and predicted class are the same, then the CN is in a non-progressive state. If the original class and predicted class are different, then CN is progressing. Finally, the performance of the model is detected by using evaluation metrics. Figure 2 showcases the architecture of the proposed study.

### 3.1 Proposed ResNet101 and LSTM for feature extraction and classification

After pre-processing, the pre-processed images are passed for feature extraction. Feature extraction is used for extracting the relevant features needed for the model. Thus, feature extraction is considered to be one of the important steps for classifying the images. By using a feature extraction mechanism, features with noise and irrelevant details are removed and aid in focusing on important aspects of the data. Furthermore, extracting and selecting aids in enhancing the interpretability of the model. Although there are various models for feature extraction, the proposed model focuses on employing the ResNet101 model for an effective feature extraction process, as the ResNet101 model is a CNN technique which is 101 layers deep, allowing it to learn rich and complex feature representation from images. This enables the ability to capture the intricate patterns and features within the images, making it suitable for extracting detailed and relevant features. Similarly, ResNet101 uses skip connections, which helps to mitigate the vanishing gradient issue and enables a swift feature extraction process. Similarly, the ResNet101 model possesses the potential to extract high-level features due to its depth and training on a diverse dataset. Owing to these factors, ResNet101 is used.

**Figure 3:**
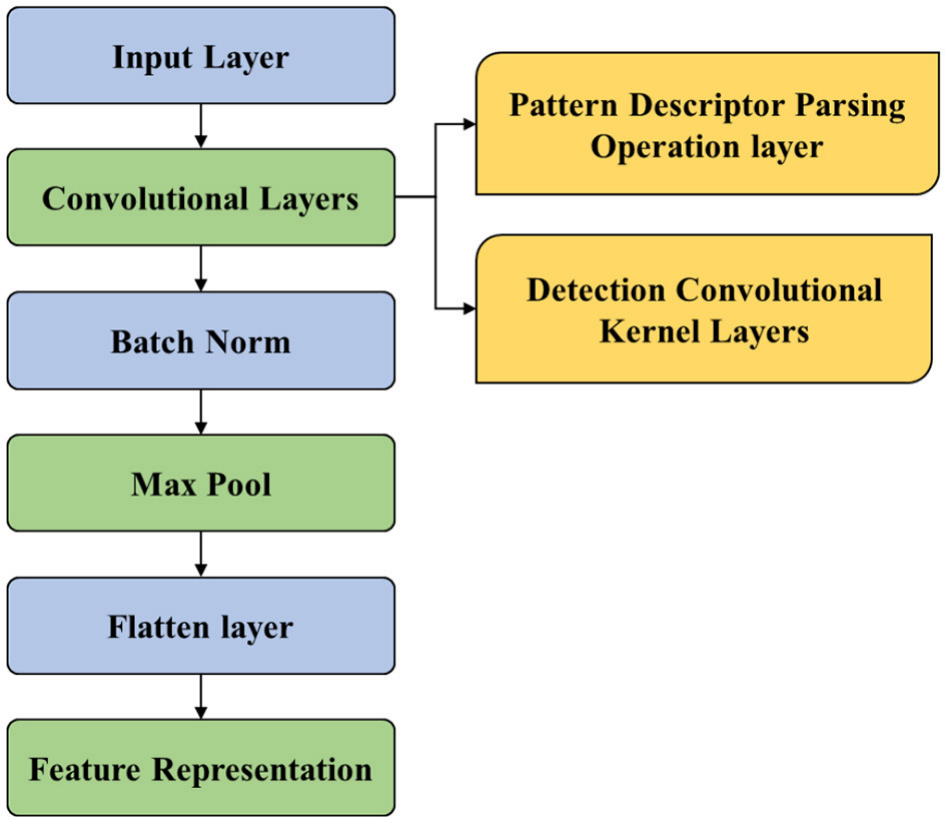
Proposed ResNet101.

Although conventional ResNet101 offers various advantages for feature extraction, certain pitfalls need to be overcome, which include the complexity of the model making it more computational and resource-intensive. This aspect of the model can lead to longer training times. Similarly, conventional ResNet101 is also susceptible to overfitting the model and interpretability of the model, making it a challenging factor for feature extraction. Thus, to overcome these drawbacks, the proposed model emphasizes using an enhanced ResNet101 model which utilizes pattern descriptor parsing operation layer function and detection convolutional kernel layer function. Hence, the proposed ResNet101 model is depicted in Figure 3.

Figure 3 showcases the process involved in the proposed ResNet101 for feature extraction. This process is carried out by sending the pre-processed features to the input layer. From the input layer, the data are forwarded to the convolutional layer. CL is considered the building block utilized for the FE process. CL encompasses a series of convolutional filters that scan input images to extract the edges, textures, and shapes. However, to enhance the ability of the feature extraction function, the PDPO layer and DCK layer are used. PDPO is employed for assigning binary codes to pixels depending on the comparison with neighboring pixels, by efficiently capturing the local texture information. Furthermore, the PDPO layer enhances the ability by considering the relationships of pixels at varying distances from the center pixels, enabling the capture of texture variations at different scales.

Therefore, PDPO layer is designed to enhance feature extraction by capturing local texture information through a binary coding mechanism. In this layer, each pixel in the input image matrix is compared with its neighboring pixels within a defined neighborhood, such as a 3 × 33 × 3 grid. For each central pixel, the layer assigns a binary code based on whether it is greater than or less than its surrounding neighbors. This process emphasizes local texture variations, allowing the model to capture subtle details that are critical for tasks such as Alzheimer's disease classification. The size of the neighborhood can be adjusted to enhance sensitivity to local features, and an optional threshold can be applied to refine the binary coding. The resulting binary feature map retains the spatial structure of the input image while reducing dimensionality, making subsequent processing more efficient. Unlike traditional convolutional layers that aggregate features over larger areas, the PDPO layer focuses on local pixel relationships, thereby improving the model's sensitivity to texture variations that might be overlooked by standard methods. Here, Equation (1) shows the process involved in PDPO.


(1) $$\begin{array}{l} C\left(a,b\right)=\left(U*V\right)\left(a,b\right)=\sum_{r}\sum_{s}U\left(a+r.b+s\right)V\left(r,s\right) \end{array}$$

where *U* is represented as input matrix image, *C* is denoted as an output feature map, and *V* is represented as the size of the filter. *a, b* denotes the enhanced input image, *r* is represented as neighboring pixel, and *s* is represented as center pixel. This input *U* is convolved with filter *V* and generates feature map *C*. Thus, the convolutional operation is denoted by U^*^V. Therefore, the convolution operation U^*^V involves multiplying corresponding elements of the filter *V* with overlapping regions of the input matrix *U*, followed by summing these products to produce a single value for each position in the output feature map *C*. This operation enables the PDPO layer to extract binary-coded features that highlight subtle texture variations critical for tasks such as Alzheimer's disease classification.

Like PDPO, DCK layer is implemented at CL for extracting the hierarchical features from the input images. The proposed DCK layer captures the discriminative effectively by sliding a tiny filer over the input image and compute element-wise multiplication between the filter and overlapping regions of the input data. This operation results in a single scalar value, which represents a feature of the input data. Therefore, DCK function predominantly aids the proposed ResNet101 model to extract hierarchal features and prevents the model from getting overfitting. Equation (2) depicts the same.


(2) $$\begin{array}{l} PDPOL_{P,R}\;\left(a_{s}\right)=\overset{r-1}{\underset{r=0}{\sum }}\mu \left(a_{r}-a_{s}\right)2^{p} \end{array}$$

where *R* is defined as the radius and distance of neighboring pixels from the center pixel. This defines the spatial extent of the neighborhood used for comparison, influencing sensitivity to local features; similarly, *P* is denoted as the number of neighboring pixels. Then, the hierarchal features are passed to batch normalization. Batch normalization is typically utilized after CL to improve training and generalization of the model by solving the internal covariance shift problem. The output from the batch normalization process is passed to the max pooling layer, which reduces the spatial dimensions of feature maps without distressing depth by introducing the translation invariance and reducing the number of learnable parameters in the succeeding layers. Eventually, the flattening layer transforms the output feature maps of the pooling layer into the 1D vector. By doing so, it helps in improving computational efficiency. Finally, the extracted features obtained are passed to the LSTM model for the classification of images as CN, MCI, and AD.

The classification is proceeded by using LSTM approach, as the LSTM model can handle complex and non-linear relationships in data, making it suitable for Alzheimer's disease classification, where the relationships between the pixels are highly intricate. Furthermore, the LSTM model is highly flexible and can be easily adapted to different input images, making the model effective for Alzheimer's disease classification.

Here, the integration of the LSTM model with ResNet101 is designed to harness the strengths of both architectures for improved feature extraction and temporal processing. In this approach, features are first extracted from the input images using the proposed ResNet101 model, with proposed PDPO and DCKL layer that capture spatial hierarchies and complex patterns. After passing through the ResNet101 architecture, the output feature maps are typically flattened to reduce their dimensionality, resulting in a fixed-length feature vector for each image. This feature vector is then prepared for input into the LSTM classification model.

**Figure 4:**
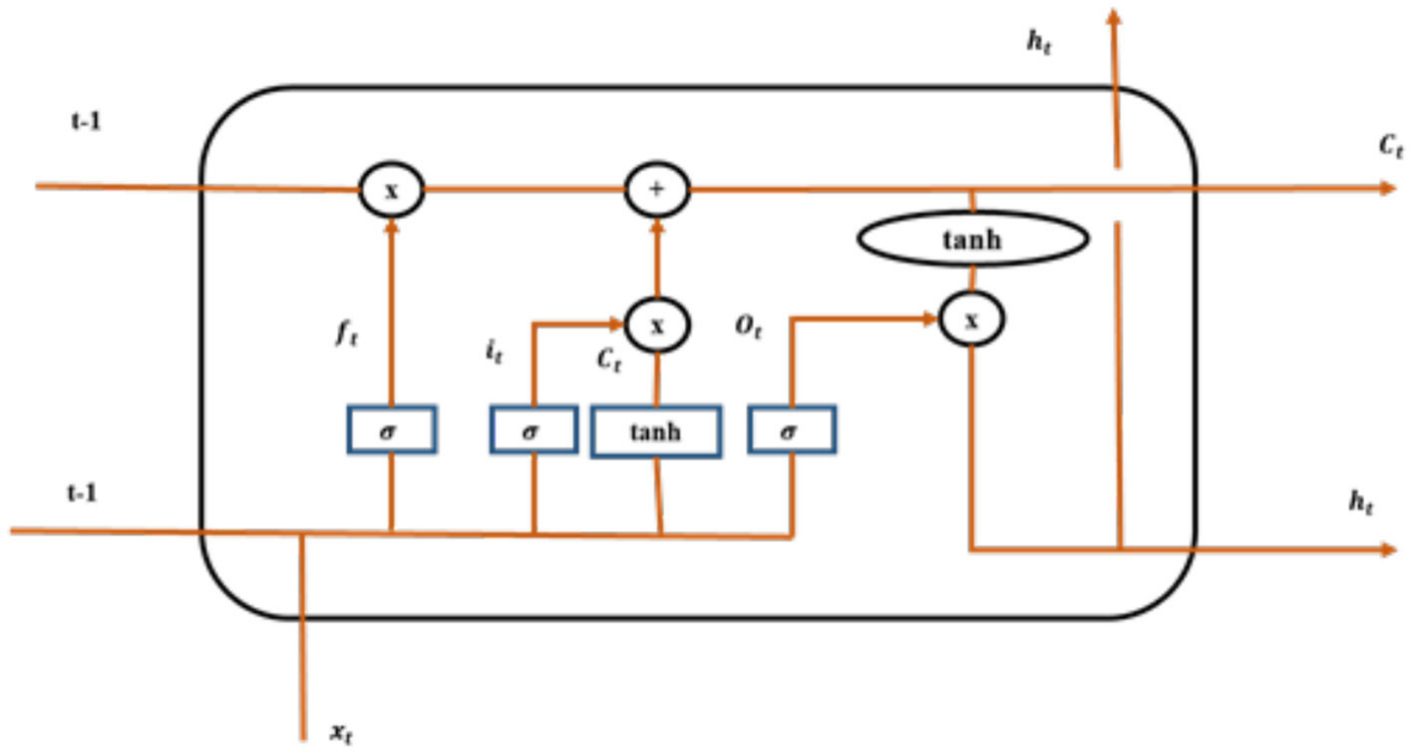
LSTM model.

To facilitate this integration, the feature vectors are organized sequentially, reflecting the temporal order of the input data, such as a series of images in a video or a sequence of frames. The LSTM is configured with specific parameters, including the number of hidden layers, the number of units in each layer, and dropout rates to prevent overfitting. Typically, the LSTM may have one or more layers with a varying number of units, depending on the complexity of the task. The output from the LSTM can be further processed to produce predictions or classifications based on the learned temporal dependencies in the data. This integration allows the model to capture both spatial features from the ResNet101 and temporal relationships through the LSTM, enhancing the overall performance in tasks such as Alzheimer's disease classification. Therefore, Figure 4 illustrates the architecture of LSTM model for classification.

In Figure 4, *f*_*t*_ is denoted as a forget gate, σ is signified as sigmoid function, *i*_*t*_ and *o*_*t*_ are denoted as the input gate and output gate, *C*_*t*_ is denoted as candidate gate, and *t*−1 is represented as cell state. Employment of LSTM model aids effectively for the classification of Alzheimer's disease. The forget gate implemented in the model is depicted in Equation (3).


(3) $$\begin{array}{l} fg_{t}=sig\left(wght\left[in_{t,}act_{t-1},C_{t-1}\right]\right)+bi_{f} \end{array}$$

In the above equation, *act*_*t*−1_ is denoted as output of the preceding block, bias vector is characterized as *b*_*f*_, input sequence is denoted by using in, *C*_*t*−1_ is represented as the previous memory block of the LSTM, *sig* is denoted as the sigmoid function, and separate weight vectors for each input are represented using *wght*. Input gate is a section, where a new memory is generated by using a trivial neural network with tanh activation function and this is depicted Equations (4) and (5).


(4) $$\begin{array}{l} i_{t}=sig\left(wght\left[in_{t,}act_{t-1},C_{t-1}\right]\right)+bi_{i} \end{array}$$

(5) $$C_{t}=f_{t}.C_{t-1}\;+i_{t}\;tanh[in_{t,}act_{t-1},C_{t-1}])+bi_{c}$$

Output gate is the section, where output generated by the current LSTM block is generated by using output gate and these outputs are estimated using Equations (6) and (7).


(6) $$\begin{array}{l} sig_{t}=sig\left(W\left[in_{t,}act_{t-1},C_{t}\right]\right)+b_{o} \end{array}$$

(7) $$\begin{array}{l} act_{t}=o_{t}.\tanh\left(C_{t}\right) \end{array}$$

Thus, the connection between the units of LSTM permits the information to cycle between adjacent time steps.

### 3.2 Determination of brain deterioration rate using GAN model

The GAN model plays a critical role in determining disease progression by generating synthetic images that simulate various stages of the disease. Once the GAN is trained, it generates images that represent both progressive and non-progressive cases. The training process involves two components: the generator, which creates synthetic images, and the discriminator, which evaluates the authenticity of these images by comparing them to real images from the dataset. In this workflow, the GAN is trained using a specific loss function, a combination of adversarial loss and additional metrics that quantify the differences between the generated and real images. The adversarial loss encourages the generator to produce images that are indistinguishable from real images, while the discriminator's loss focuses on correctly classifying real vs. generated images. A common choice for the loss function in GANs is the binary cross-entropy loss, which measures the performance of the discriminator in distinguishing real images from fake ones.

After training, the model is tested using images generated by the GAN. By analyzing the characteristics of these synthetic images in comparison with the original images, the model can identify patterns indicative of disease progression. This approach allows for a nuanced understanding of the disease's trajectory as the GAN-generated images can reflect subtle changes that may not be easily observable in the original dataset. The ability to compare these generated images with actual clinical cases enhances the model's capacity to distinguish between progressive and non-progressive cases, ultimately contributing to more accurate predictions regarding disease progression.

Thus, GAN model is used in the proposed model for analyzing and predicting the progression of Alzheimer's disease based on original class and predicted class by generating new images based on the patient's image data. Once trained, the GAN model can generate synthetic data which represent different stages of Alzheimer's disease progression. Therefore, by comparing the original class with the predicted class, the progression of AD can be identified. Here, both the generator and discriminator were optimized using the Adam optimizer with a learning rate of 0.0002 and a beta1 value of 0.5. This choice of optimizer helps in achieving faster convergence and stability during training. The training process involved alternating updates between the generator and discriminator, ensuring that each model learns effectively from the other's performance.

**Figure 5:**
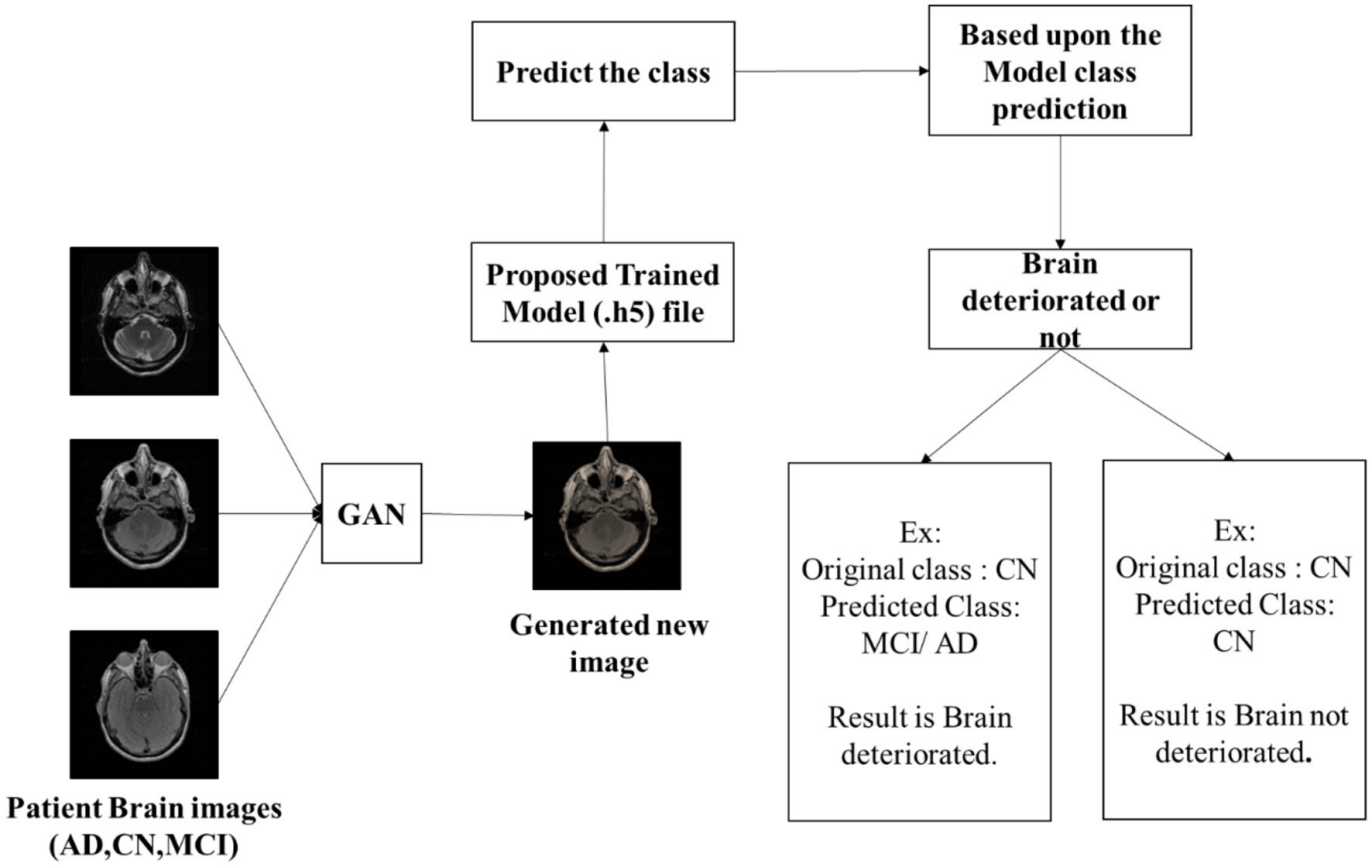
GAN model.

Thus, the process carried out by the GAN model for determining brain deterioration rate is depicted in Figure 5.

Initially, the model was trained with accuracy of 99%. In general, a GAN model comprises a generator and a discriminator where the generator network in the GAN model generates the synthetic data samples and the discriminator network evaluates the generated samples of data and the original data samples to distinguish between progressive and non-progressive classes. In the GAN model, the generator generates an image from the original class, whereas the discriminator generates other images from the dataset. If the image generated by the discriminator is progressive, such as CN, MCI, and AD, then the disease is identified to be progressive. If not the disease, it is identified to be non-progressive.

If the original class is CN and the predicted class obtained by the proposed model is AD, it is noted that the brain deterioration rate is in the progressive state as the original class is CN, whereas the predicted class appears to be in progressive nature by predicting AD. Conversely, if the original class is MCI and the predicted class is MCI as well, then there is no progression in terms of brain deterioration rate. Eventually, the GAN model utilizes a loss function for measuring the difference between ground truth labels and predicted classes. This loss function guides the training process for minimizing the errors in classifying the progressive and non-progressive nature precisely. The advantages of employing the GAN model in the proposed framework include the following:

- Detection of progressive and non-progressive Alzheimer's disease.
- Identification of brain deterioration rate can help in preventing adverse consequences.

Therefore, Table 1 shows the outcome obtained by GAN model for brain deterioration rate.

**Table 1:** Brain deterioration rate.

**Original image class**	**Predicted class**	**Brain deterioration rate**
CN	CN	Brain not deteriorated
CN	MCI	Brain deteriorated
CN	AD	Brain deteriorated
MCI	AD	Brain deteriorated

Table 1 shows the brain deterioration rate. Here, it was projected that there is brain deterioration when the original image class and predicted class are the same. That is, when the original class is CN and the predicted class is CN, it means that the brain is not deteriorated. However, if the original class and reduced class are different, it is depicted that the brain is deteriorated. Therefore, the GAN model is used for detecting brain deterioration rates.

The subsequent section deals with the results obtained using the proposed model by assessing the efficacy of the proposed framework using metrics such as accuracy, recall, F1 score, and precision value.

## 4 Result and discussion

Result and discussion section primarily involves depicting the outcome of the proposed model post-deployment for the classification of Alzheimer's disease as CN, MCI, and AD. Hence, subsequent section discusses about metrics involved, EDA, and performance analysis of the model.

### 4.1 Dataset description

The proposed study utilizes two different datasets for AD multiclass classification such as ADNI (Alzheimer's disease neuroimaging initiative dataset) and OASIS dataset.

#### 4.1.1 Creation and collection of data

The dataset is created by gathering subject information and image information, in which the subject information consists of subject ID, research group, age, research group, weight (in Kg), and other aspects. Similarly, in image information, parameters such as modality (DTI, MRI, PET, Path, and fMRI), image description, image ID, weighting, slice thickness, and acquisition plane are considered.

##### 4.1.1.1 ADNI dataset

The clinical dataset comprises of detailed clinical information from each subject which includes extensive patient measurements such as MRI data. It encompasses data from North America male and female individuals, with a total of 502 attributes collected from 1737 participants. Specifically, the dataset includes data from 1453 male patients and 1074 female patients. Table 2 shows the sample patient ID with MRI counts.

**Table 2:** Sample patient ID for ADNI.

**Sample patient ID**	**MRI count**
132_S_0339	3
035_S_6947	3
130_S_6319	3
023_S_0331	3
132_S_0339	3
035_S_6947	3
130_S_6319	3
023_S_0331	3
023_S_0030	3
128_S_0216	5
116_S_6624	5
127_S_0260	5
041_S_0282	5
127_S_0397	6

Table 2 depicts MRI count taken by different patients along with patient ID. Patient ID with 132_S_0339 has taken MRI count of 3, ID with 130_S_6319 has taken MRI count of 3, and 5 numbers of MRI have been taken by patient ID with 116_S_6624, 128_S_0216, 127_S_0260, and 041_S_0282. Similarly, 6 MRI has been taken by patient with ID 127_S_0397.

Similarly, Table 3 depicts samples of patients who has taken MRI. Here, 92 patients have taken 3 MRIs, 12 patients have taken 4 MRI, 4 patients have taken 5 MRI, and so.

**Table 3:** MRI count and patient count for ADNI.

**ADNI MRI**	**MRI count**	**Patient count**
	4	12
	3	92
	5	4
	6	1
	14	1
	12	2

##### 4.1.1.2 OASIS dataset

The dataset provides neuroimaging and related clinical data, encompassing neuroimaging data across the genetic spectrum, and cognitive and demographic factors for researchers studying Alzheimer's disease. Specifically, data from 1,317 male patients and 1,911 female patients have been collected for research purpose.

### 4.2 Performance metrics

#### 4.2.1 Accuracy

The accuracy is claimed as the calculation of total accurate classification. The accuracy range is premeditated by using Equation (8),


(8) $$\begin{array}{l} Acc=\frac{TN\;+\;TP}{TN\;+FN\;+TP\;+FP} \end{array}$$

where *TN* is represented as true negative, and *FN* is represented as false negative; similarly, true positive and false positive are denoted by using *TP* and *FP*.

#### 4.2.2 Precision

The precision is considered by determining the accurate classification count. It is calculated through indecorous classification. The precision is estimated by using Equation (9),


(9) $$\begin{array}{l} precision=\frac{TP}{FP\;+\;TP} \end{array}$$

#### 4.2.3 F-measure

The F1 score is represented as the weighted harmonic-mean value of precision and value of recall, and Equation (10) is defined as the formula employed for determining F1-Score,


(10) $$\begin{array}{l} F1\;-\;score=2\;\times \;\frac{R\times P}{R+P} \end{array}$$

where *P* is denoted as precision, and *R* is denoted as recall.

#### 4.2.4 Recall

The recall is indicated as the reclusive of the production metric that assesses the total of correct positive categories made out of all the optimistic classes. Equation (11) shows the mathematical model for recall,


(11) $$\begin{array}{l} Recall=\frac{TP}{FN\;+\;TP} \end{array}$$

### 4.3 System configuration

Experimental setup including hardware and software requirements for implementing proposed methodology is depicted in Table 4.

**Table 4:** System configuration.

**Techniques**	**Tools and requirements**
MRI image data	MRI datasets(brain)
Hardware requirements	•Adequate computational properties: CPU and GPU.
Software requirements	•Image processing libraries and frameworks such as OpenCV (Open Source Computer Vision Library) and Tensor Flow. •Python or other programming languages
Visualization tools	Matplotlib visualization of volumetric medical images.
Data pre-processing tools	•DICOM (Digital Imaging and Communications in Medicine) format conversion python code. •Image registration and normalization software

### 4.4 EDA

**Figure 6:**
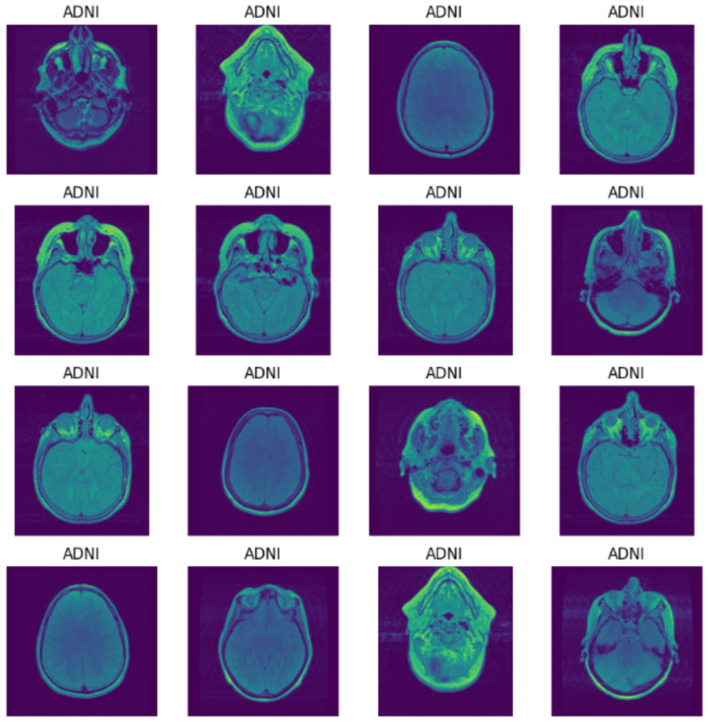
MRI of ADNI.

**Figure 7:**
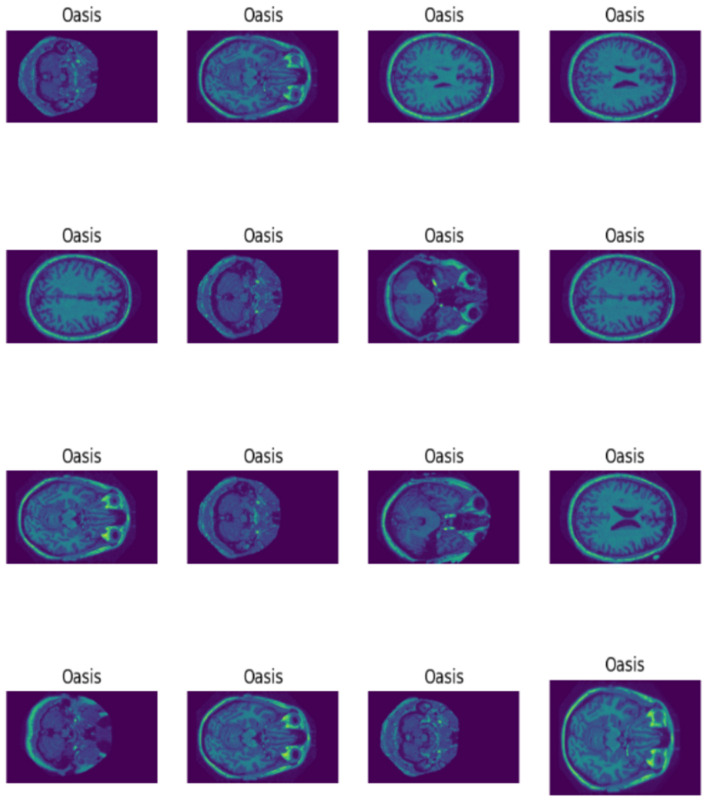
MRI of OASIS.

EDA plays a crucial role in comprehending the insights, characteristics, and patterns of the data in the dataset. Therefore, EDA for Alzheimer's disease uncovers significant relationships and trends in terms of biomarkers, risk factors, and patterns which may contribute toward the progression, diagnosis, and treatment of the disease. Moreover, EDA also aids in detecting data quality issues, missing values, and outliers to ensure the reliability and accuracy of the model. Thus, Figures 6, 7 show the MRI scans of ADNI dataset and OASIS dataset.

**Figure 8:**
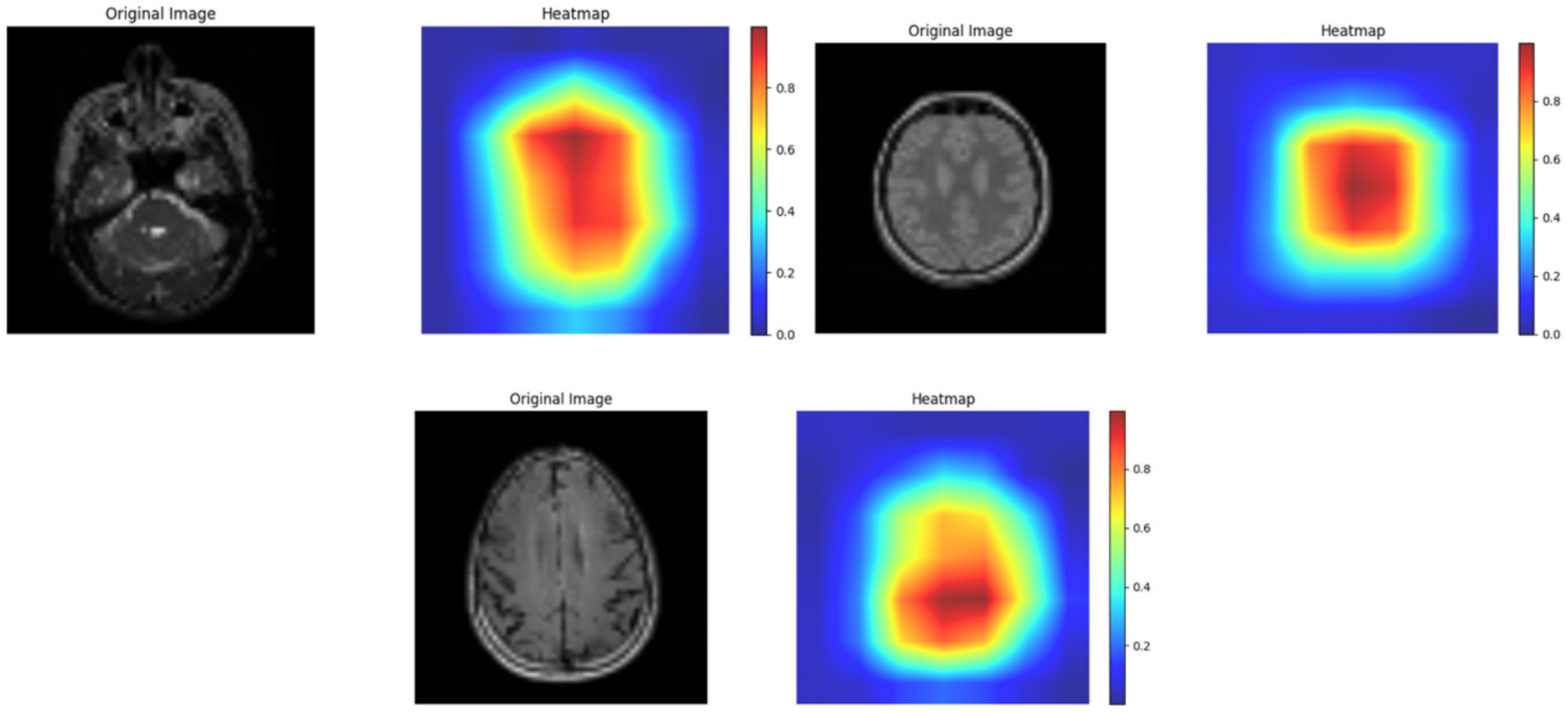
Heatmap for ADNI dataset.

**Figure 9:**
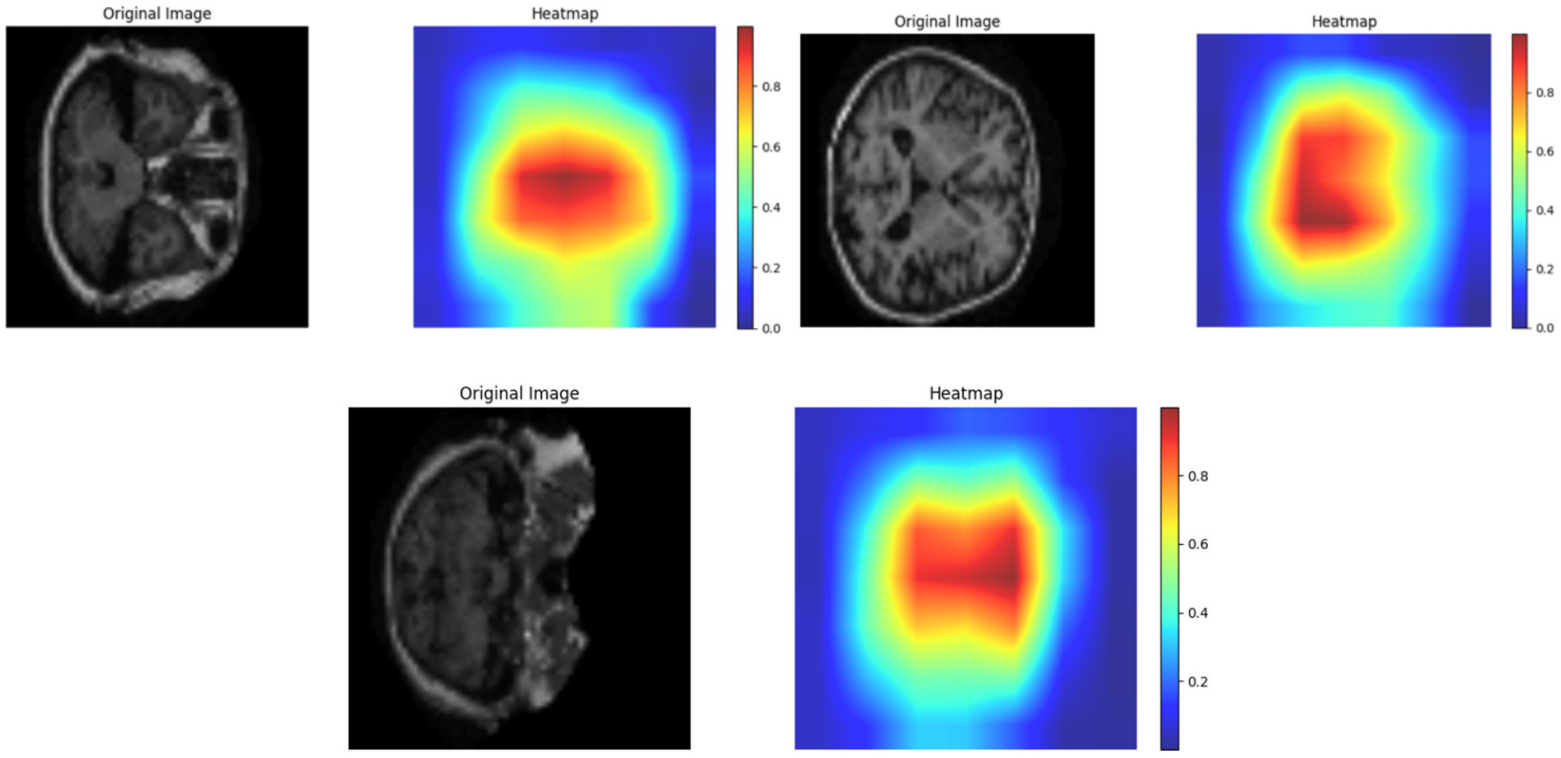
Heatmap for OASIS dataset.

Thus, MRI scans of ADNI dataset and OASIS dataset are illustrated in Figures 6, 7 from different angles. Similarly, heatmap for ADNI and OASIS dataset is depicted in Figures 8, 9.

Heatmap is used for exploring the datasets and aids in detecting the patterns and trends with varying colors. This heatmap assists in highlighting the area of outliers or concentration. Each ROI can be denoted by a heatmap, showing the variations in intensity which corresponds to different measurement. Thus, Figure 8 showcases the heatmap of ADNI dataset, and Figure 9 demonstrates heatmap of OASIS dataset.

### 4.5 Performance analysis

The performance of the proposed model is depicted in the subsequent section, where the performance of the model is analyzed using different metrics such as model accuracy, model loss, and confusion matrix for both the ADNI and Oasis datasets.

**Figure 10:**
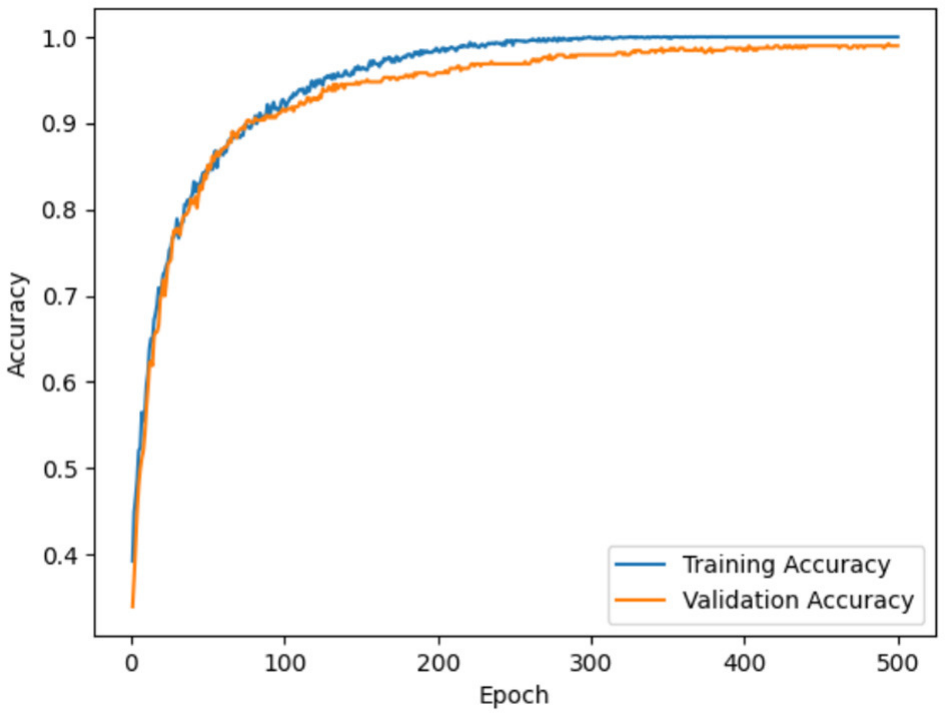
Model accuracy for ADNI.

**Figure 11:**
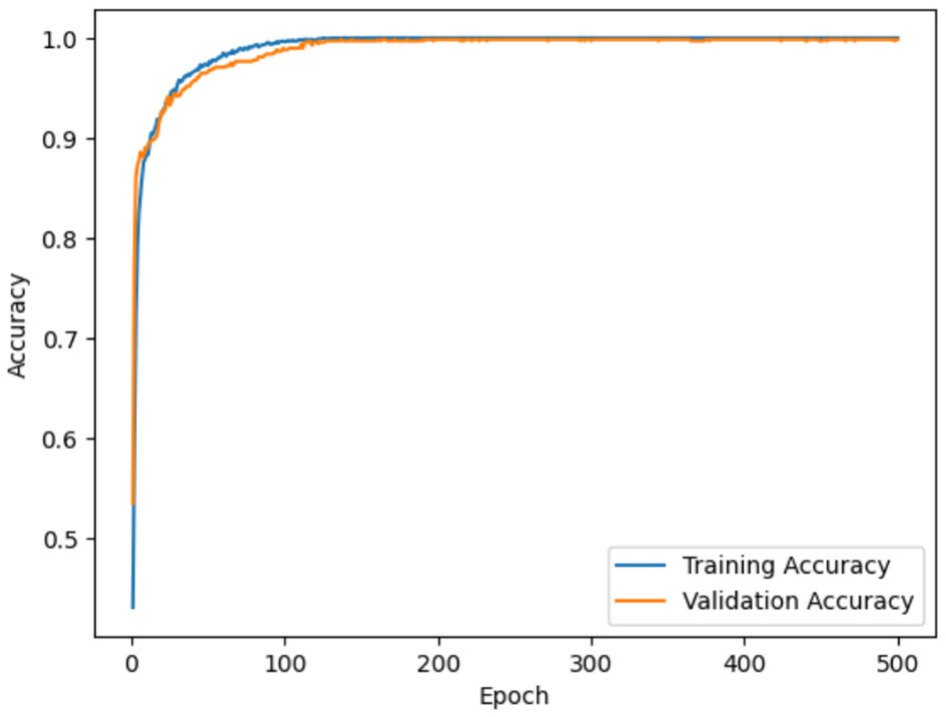
Model accuracy for OASIS.

Model accuracy for ADNI and OASIS datasets using the proposed model is portrayed in Figures 10, 11. Model accuracy graph is defined as the visual representation of how the accuracy of the model changes over time or epochs during the training process. X-axis denotes the number of epochs, and Y-axis denotes the accuracy of the model. Thus, Figures 10, 11 show the model accuracy graph for the ADNI and OASIS datasets.

The model accuracy for the ADNI dataset is depicted in Figure 10, in which the blue line represents the training accuracy and the orange line represents the validation accuracy. Training accuracy refers to the accuracy of the model on the training dataset during the training process. This indicates the ability of the model to predict the correct output for the data it was trained on. Validation accuracy denotes the accuracy of the model on a separate validation dataset for evaluating the model, on how well the model generalizes to unseen and new data. In figures, training accuracy is more than validation accuracy. This showcases that the model is learning patterns present in the training data effectively. Similarly, model loss for ADNI and OASIS is portrayed in Figures 10, 11.

**Figure 12:**
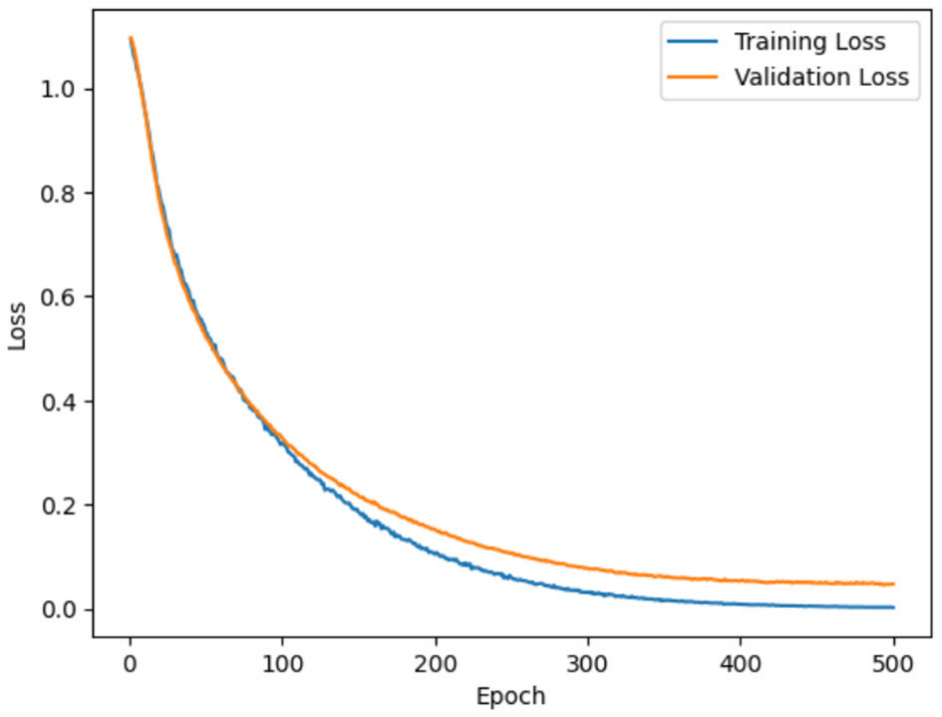
Model loss for ADNI.

**Figure 13:**
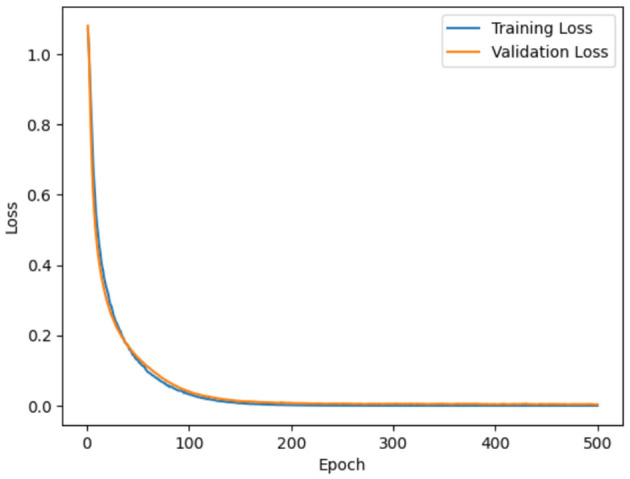
Model loss for OASIS.

Model loss using ADNI dataset and OASIS dataset is demonstrated in Figures 12, 13. Model loss refers how well the proposed model performs during training. In model loss, training and validation losses are examined, where training loss is denoted as the error between the actual or predicted output on the training dataset. The primary objective is to minimize the training loss by optimizing the parameter of the model. Similarly, validation loss is the differences between actual or predicted output on a separate validation dataset which was utilized during training process. From figures, it can be clearly observed that both validation and training losses decrease when model goes through multiple epochs of training. This showcases that proposed model is learning to make better predictions.

**Figure 14:**
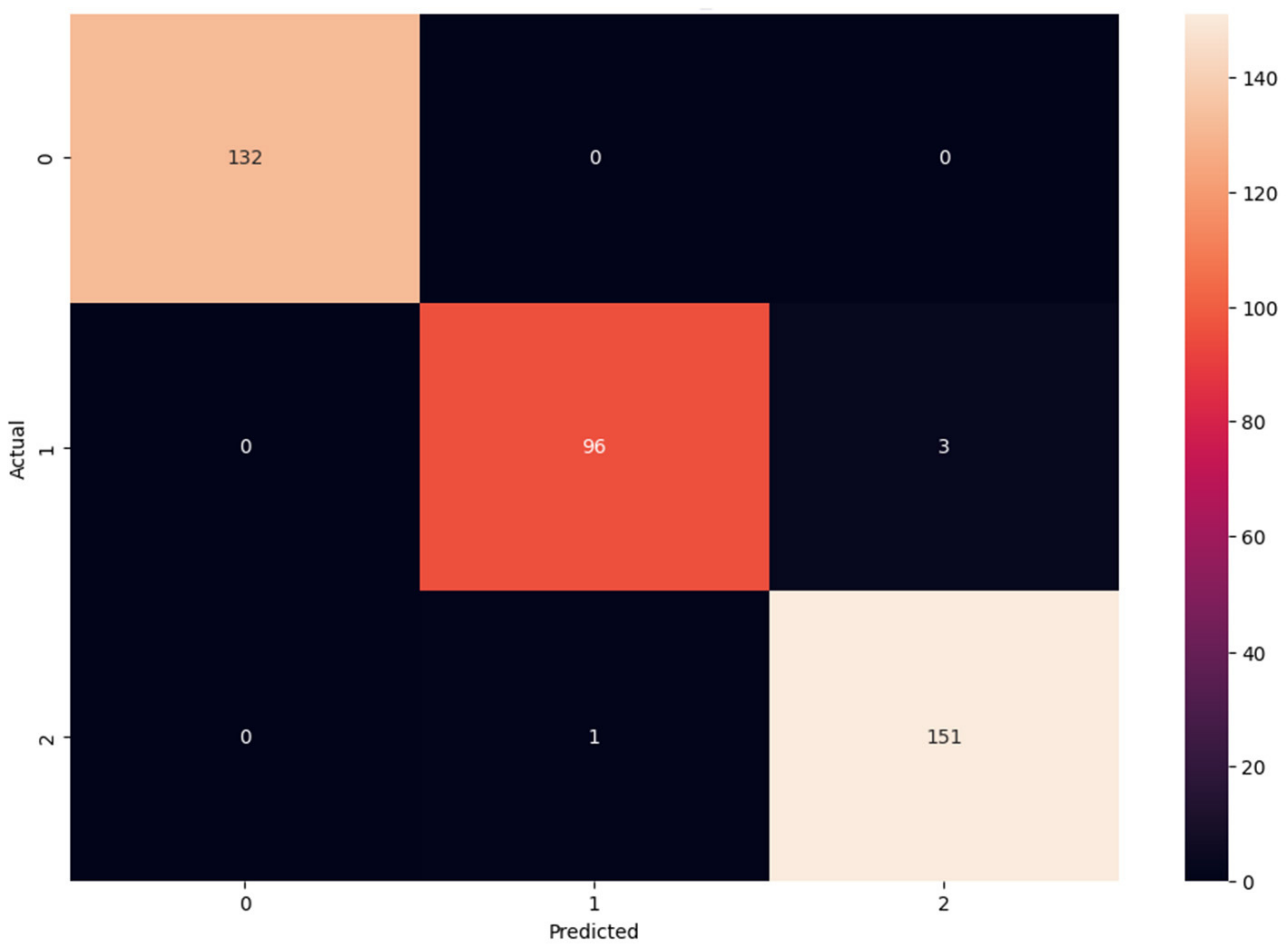
Confusion matrix for ADNI.

Like model accuracy and model loss, confusion matrix is an important assessing the performance of the proposed framework for multiclass classification of Alzheimer's disease. Confusion matrix displays number of correct classifications and misclassifications by the model compared to the actual outcomes in the dataset. In addition, row in the matrix denotes the actual class labels and column in the matrix denotes the predicted class labels. Hence, confusion matrix for ADNI dataset is denoted in Figure 14.

**Figure 15:**
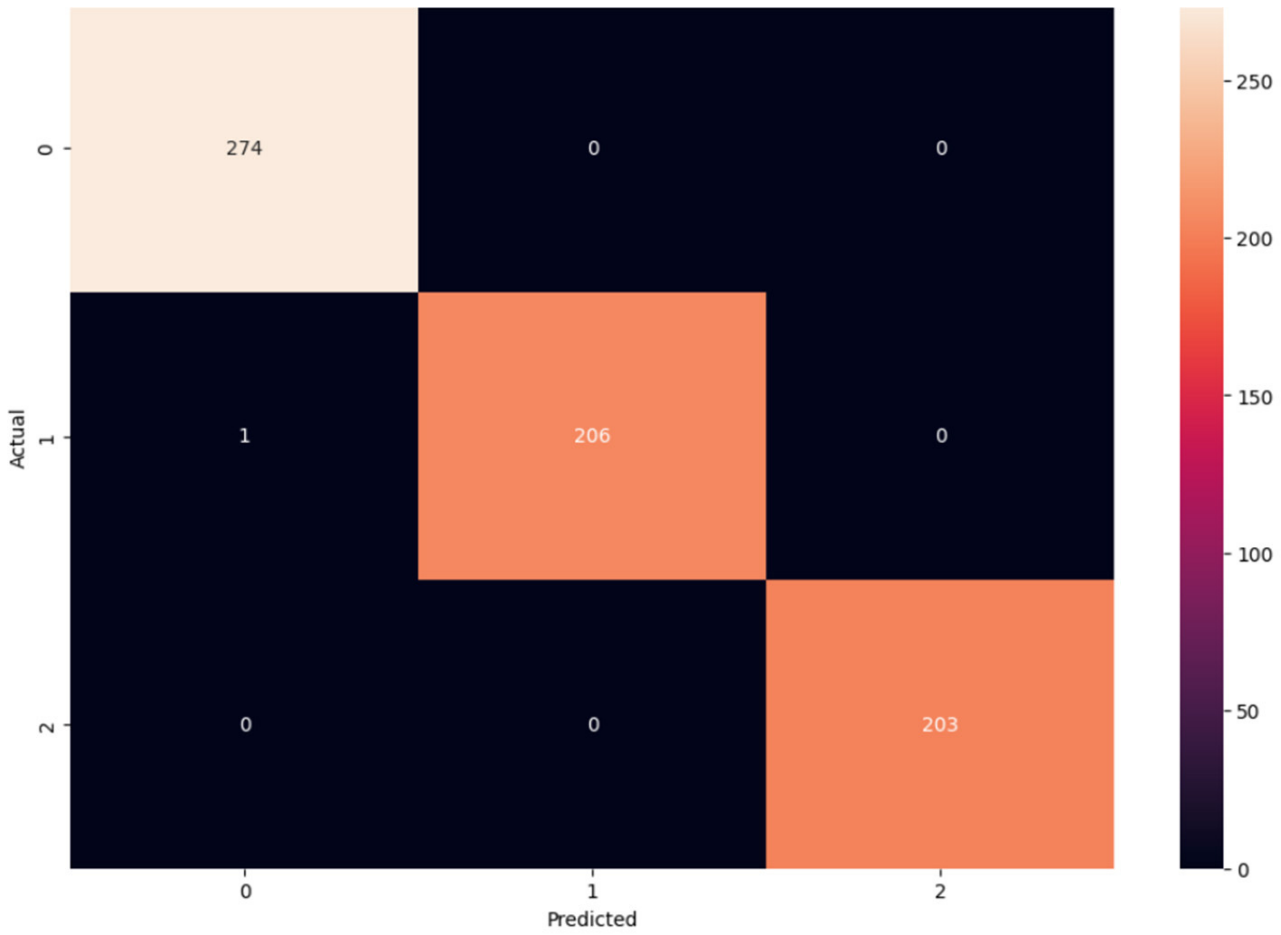
Confusion matrix for OASIS.

In Figure 14, confusion matrix for ADNI is depicted. Here, the correct classifications and misclassifications are represented in which misclassification is denoted in black color and correct classification for AD, CN, and MCI is depicted, where AD form comprises of higher correct predictions, in which the correct predictions for AD is 132, CN is 96, and MCI is 151. Similarly, confusion matrix for OASIS is illustrated in Figure 15.

Here, the confusion matrix for proposed model using OASIS is depicted in Figure 15, where correct classifications and misclassifications are represented. The correct classification for AD is 274, CN is 206, and MCI is 151. Therefore, from the experimental results, it can be observed that proposed model is capable of producing effective outcome which is essential for classification of Alzheimer's disease. Like confusion matrix, other metrics are also used for gauging the efficacy of the proposed study, which includes accuracy of the model, precision, F1 score, and recall rate. Therefore, Table 5 showcases the metric value obtained by the proposed study.

**Table 5:** Performance metrics.

**MRI images**	**Accuracy**	**Precision**	**Recall**	**F1-score**
ADNI	0.9932	0.99	0.99	0.99
OASIS	0.9985	0.99	0.99	0.99

Table 5 depicts the metrics obtained by the proposed study for both ADNI and OASIS datasets. Here, the proposed model using ADNI dataset obtains accuracy of 0.9932%, precision of 0.99%, recall rate of 0.99%, and F1 score of 0.99%. Similarly, proposed model using OASIS dataset obtains accuracy value of 0.9985%, precision value of 0.99%, recall rate of 0.99%, and F1 score of 0.99%.

**Figure 16:**
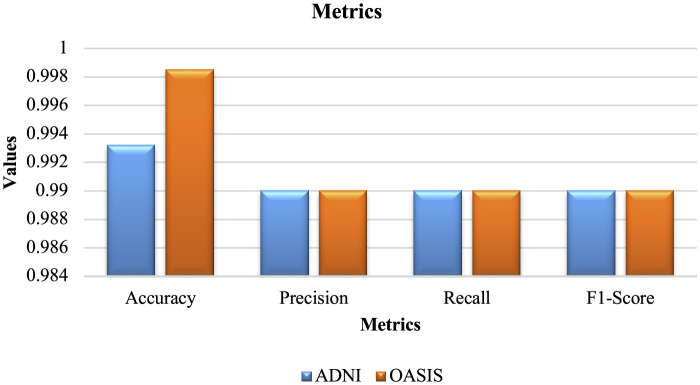
Graphical representation.

The graphical representation of Table is portrayed in Figure 16.

### 4.6 Statistical analysis

Statistical outcome using proposed model is demonstrated in the Table 6.

**Table 6:** Statistical table.

**Test**	**Values**
*t*-statistic	34.4585
*p*-value	5.9389e-33
Cohen's d	11.9870

The provided statistical values of t-statistic of 34.4585, *p*-value of 5.9389e-33, and Cohen's d of 11.9870 indicate an exceptionally strong effect size and highly significant results in the context of Alzheimer's disease classification. The t-statistic reflects a robust difference between groups, while the *p*-value confirms that this difference is extremely unlikely to be due to chance, surpassing conventional thresholds for statistical significance. Cohen's d, representing the effect size, indicates an extraordinarily large magnitude of difference between the compared groups, which is rare in clinical studies. Such values imply that the tested variable or method has a profound ability to distinguish between classifications, such as Alzheimer's disease vs. normal controls or other subgroups such as mild cognitive impairment (MCI). This level of statistical evidence strongly supports the reliability and clinical applicability of the classification approach, potentially aiding in early diagnosis or targeted intervention strategies for Alzheimer's disease.

Although the proposed model has delivered better outcome for classification of Alzheimer's disease, it is important to compare the proposed study with existing models; however, the dataset used in the model is a real time dataset; thus, external comparison is not feasible due to the implementation of real time data. However, from the analytical outcome, it can be identified that proposed study has delivered better outcome for multiclass classification of AD.

## 5 Discussion

Existing study has used Deep-CNN model for classification of AD. The model is fine-tuned to identify the subtle patterns and anomalies within the scans linked to AD. However, the finding obtained by the Deep-CNN is 96.64% of accuracy (36). Similarly, 2D and 3D CNN models (37) are explored in the study for AD classification. However, the accuracy outcome obtained by 2D-CNN model was 91.29% and 3D-CNN model was 91.07%. Moreover, classification of AD is carried out in the study based on ConvNets (38) using MRI images. However, the accuracy rates of classifications have reached up to 97.65% for AD/MCI and 88.37% for MCI/normal control. In addition, CNN is based on DenseNet Bottleneck-Compressed architecture (39) for AD diagnosis using MR images. The proposed model classified the input into five different categories, namely, CN, EMCI, MCI, LMCI, and AD, with an average accuracy of 86%. Thus, when compared to all these models, the accuracy obtained by the proposed framework is superior and effective as it gained 99.32% for ADNI dataset and 99.85% for OASIS dataset. This is due to the implementation of proposed ResNet for feature extraction and LSTM for classification.

## 6 Conclusion

The proposed research study delivered proficient results for the multiclass classification of AD as CN, MCI, and AD. Better performance was obtained for AD classification primarily due to the incorporation of effective AI approaches such as ResNet101 and LSTM. The proposed ResNet101 model used DKCL and PDPO layer for extracting relevant features needed for the proposed model. PDPO was employed to assign binary codes to pixels depending on the comparison with neighboring pixels, by efficiently capturing the local texture information, and the DCK layer captured the discriminative effectively by sliding a tiny filter over the input image and computing element-wise multiplication between the filter and overlapping regions of the input data. Implementation of these proposed functions in the proposed ResNet101 model aided in extracting relevant features needed for the model. Eventually, the extracted features were passed to the LSTM model for the classification of Alzheimer's disease as CN, MCI, and AD. In addition, the proposed research focused on employing the GAN model to find whether Alzheimer's disease is progressive or non-progressive by distinguishing the original class from the predicted class. Incorporation of the proposed model delivered a better accuracy rate of 0.9932 and 0.9985 for both ADNI and OASIS datasets.

In the future, different DL-based algorithms can be used for more advanced AD prediction. Employment of the GAN model is considered to be one of the major highlights of the proposed research study. However, this can be further developed in future study in terms of detecting brain deterioration rates for various classes. In addition, the integration of multi-modal data sources such as MEI, PET scans, and clinical biomarkers can be explored to assess the model's performance over time and to improve predictive accuracy. Thus, the combination of GANs and multi-modal data integration could pave the way for more sophisticated and accurate tools for early detection, prognosis, and management of Alzheimer's disease.

## Data Availability

The original contributions presented in the study are included in the article/supplementary material, further inquiries can be directed to the corresponding author.
